# *MATSAS*: a small-angle scattering computing tool for porous systems

**DOI:** 10.1107/S1600576721000674

**Published:** 2021-03-18

**Authors:** Amirsaman Rezaeyan, Vitaliy Pipich, Andreas Busch

**Affiliations:** a Lyell Centre, Institute of GeoEnergy Engineering, Heriot-Watt University, Research Avenue South, EdinburghEH14 4AS, United Kingdom; b Heinz Maier-Leibnitz Zentrum (MLZ), Forschungszentrum Jülich GmbH, Jülich Centre for Neutron Science (JCNS), Lichtenbergstrasse 1, Garching85748, Germany; ANSTO, Kirrawee DC, Australia

**Keywords:** *MATSAS*, small-angle scattering, polydisperse spherical model, porous media, computer programs

## Abstract

*MATSAS* is a script-based MATLAB program for analysis of X-ray and neutron small-angle scattering (SAS) data obtained from various facilities. The program has primarily been developed for sedimentary rock samples but is equally applicable to other porous media. *MATSAS* imports raw SAS data from .xls(x) or .csv files, combines small-angle and very small angle scattering data, subtracts the sample background, and displays the processed scattering curves in log–log plots. *MATSAS* uses the polydisperse spherical (PDSP) model to obtain structural information on the scatterers (scattering objects); for a porous system, the results include specific surface area (SSA), porosity (Φ), and differential and logarithmic differential pore area/volume distributions. In addition, pore and surface fractal dimensions (*D*_p_ and *D*_s_, respectively) are obtained from the scattering profiles. The program package allows simultaneous and rapid analysis of a batch of samples, and the results are then exported to .xlsx and .csv files with separate spreadsheets for individual samples. *MATSAS* is the first SAS program that delivers a full suite of pore characterizations for sedimentary rocks. *MATSAS* is an open-source package and is freely available at GitHub (https://github.com/matsas-software/MATSAS).

## Abstract 2

*MATSAS* analyses X-ray and neutron small-angle scattering data obtained from porous systems. *MATSAS* delivers a full suite of pore characterizations, including specific surface area, porosity, pore size distribution and fractal dimensions.

## 1. Introduction

Small-angle scattering (SAS) of neutrons and X-rays (SANS and SAXS, respectively) is widely used for the nondestructive study of the low-resolution structure of natural and engineered systems, including sedimentary rocks, biological macromolecules, composite nanomaterials and polymers on length scales between ångströms and micrometres in a single or combined experiment (Feigin & Svergun, 1987 ▸; Binder *et al.*, 2000 ▸; Zemb & Lindner, 2002 ▸; Radlinski, 2006 ▸; Borsali & Pecora, 2008 ▸; Anovitz & Cole, 2015 ▸; Melnichenko, 2015 ▸; Fritzsche *et al.*, 2016 ▸). Advances in SAS instrumentation, such as neutron and high-flux X-ray synchrotron beamlines, have significantly increased the use of SANS and SAXS experiments (Melnichenko, 2015 ▸; Zemb & Lindner, 2002 ▸; Heenan *et al.*, 1997 ▸). With the availability of these technologies, modern instruments can provide high-quality data in time- or space-resolved experiments or measurements under various physical and chemical conditions, such as temperature, pressure, humidity *etc.* (Konarev *et al.*, 2006 ▸; Schrank *et al.*, 2020 ▸). The theoretical and methodological developments obtained over the past few decades have allowed the retrieval of structural information from SAS patterns to address questions revolving around the size, shape, distribution and orientation of scatterers (scattering objects) (Konarev *et al.*, 2006 ▸; Petoukhov *et al.*, 2012 ▸).

**Figure 1:**
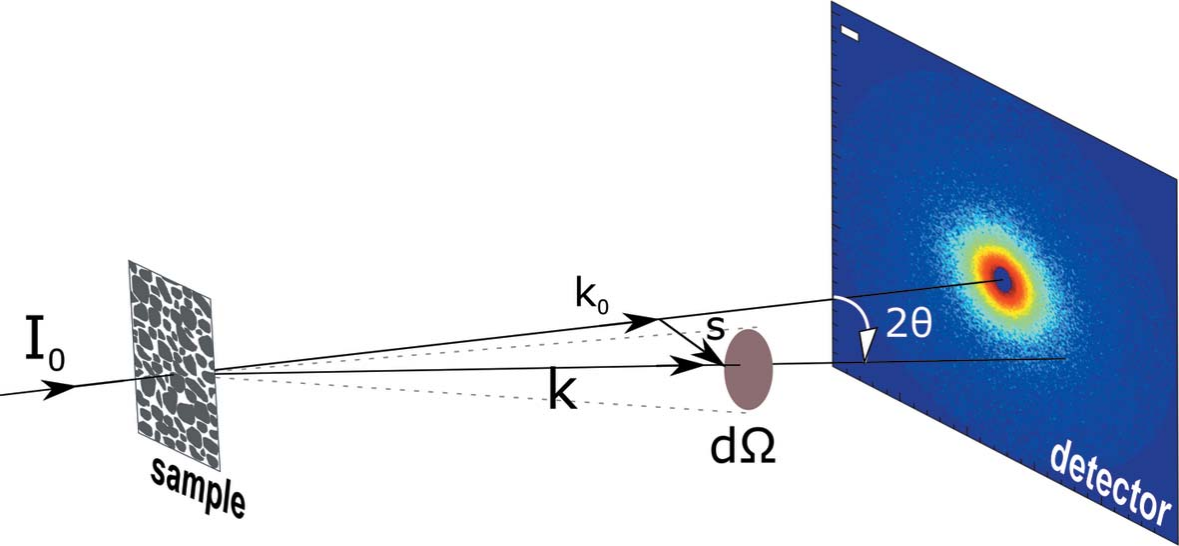
The schematic principle of a SAS experiment.

Neutron and X-ray scattering techniques complement each other, but neutrons and X-rays are different in their charge, energy and interaction with matter, which makes each technique subject to its own experimentation type and/or sample type (Binder *et al.*, 2000 ▸; Zemb & Lindner, 2002 ▸; Melnichenko, 2015 ▸). Fig. 1 ▸ illustrates a pinhole SAS experiment. Neutrons or X-rays are collimated and monochromated towards the sample, inside which a neutron or photon is elastically scattered from its wavevector **k**_0_ into a state with wavevector **k** under a scattering angle 2θ. The magnitude of a wavevector relates to its wavenumber, which is |**k**| = |**k**_0_| = *k* = 2π/λ for elastic scattering, where λ is the neutron or X-ray wavelength. The intensity of the scattered radiation d*I* is therefore measured in the direction **k** as a function of the momentum transfer (the convention **s** = |**k** − **k**_0_|) or the scattering vector **Q**. The magnitude of the scattering vector is given by *Q* = 4πsinθ/λ, from which it follows that **Q** = 2π**s**, where *s* = 2sinθ/λ (Radlinski, 2006 ▸; Melnichenko, 2015 ▸).

The incident flux of the scattering objects is denoted by Φ_0_, *i.e.* Φ_0_ = *I*_0_/*A*, where *I*_0_ is the incident intensity (neutrons or X-rays per second) and *A* is the beam cross-sectional area at the sample position (Radlinski, 2006 ▸). The scattered intensity monitored in the solid-angle element dΩ targeted by *Q* can be expressed as 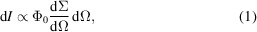
where dΣ is the elemental scattering cross section. The quantity dΣ/dΩ is called the differential cross section of scattering (Radlinski, 2006 ▸). The aim of SAS experiments is to determine volume-averaged information on the spatial distribution of the scattering length density (neutrons) or electron density (X-rays) in the sample from the measured dΣ/dΩ as a function of the scattering vector magnitude *Q*, thus 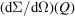 or *I*(*Q*) (Melnichenko, 2015 ▸).

For a wide range of substances, SAS data for hard and soft matter can generally be interpreted accurately using a two-phase approximation (Melnichenko, 2015 ▸). In this approximation, the scattering volume is viewed as being composed of above-molecular-size phases, each characterized by one of two possible values of the physical property that provides the scattering contrast (Δρ*). For instance, for porous media, these two phases are the solid matrix (phase 1) and the pore space (phase 2) (Radlinski, 2006 ▸). The two-phase approximation is a simplification inherent in the SAS method and has been implicitly or explicitly employed for many years. As such, the general expression of the scattering cross section can be expressed as
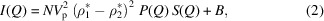
where *N* is the number density of scatterers *N*_p_ per unit volume, *V*_p_ is the volume of the scatterers, and 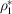 and 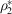 are the scattering length/electron density of phase 1 and phase 2, respectively. *B* is the sample background, accounting for scattering in the high-*Q* limit. The high-*Q* background originates from (i) *Q*-independent incoherent scattering caused by hydrogen atoms in organic matter and/or water, and (ii) *Q*-dependent coherent scattering resulting from microscopic inhomogeneities (*e.g.* small pores in the rock matrix; Bahadur *et al.*, 2015 ▸; Blach *et al.*, 2020 ▸). *P*(*Q*) is the form factor which describes the size and shape of the scatterer. There are analytical expressions for the form factor for simple geo­metrical objects like spheres, cylinders, discs or parallelepipeds (Melnichenko, 2015 ▸). *S*(*Q*) is the structure factor and contains information about the spatial distribution of the scatterers. The structure factor represents the modification of the intensity due to the spatial correlation of the scatterers (Fritzsche *et al.*, 2016 ▸), where the positions of the scatterers are frozen in time and space in solid porous materials (Melnichenko, 2015 ▸). In soft-matter systems, the interaction potential between scatterers is also taken into consideration (Melnichenko, 2015 ▸). Form and structure factors need to be specified to determine the structural information in the scattering curves.

Several SAS programs have been developed in different laboratories that consider various data processing and manipulation methods, fitting models, and form and structure factors to characterize the structure of the scatterers (Table 1 ▸). Recognizing the increasing application of SAS data to analyse the pore structure of sedimentary rocks, especially low-permeability rocks such as coal and mudrocks or gas shales (Radlinski, Ioannidis *et al.*, 2004 ▸; Radlinski, Mastalerz *et al.*, 2004 ▸; Radliński *et al.*, 2009 ▸; Mares *et al.*, 2012 ▸; Clarkson *et al.*, 2012 ▸; Mastalerz *et al.*, 2012 ▸; Melnichenko *et al.*, 2012 ▸; Bahadur *et al.*, 2014 ▸, 2015 ▸; Anovitz *et al.*, 2015 ▸; Leu *et al.*, 2016 ▸; Busch *et al.*, 2017 ▸, 2018 ▸; Anovitz & Cole, 2018 ▸; Sakurovs *et al.*, 2018 ▸; Vishal *et al.*, 2019 ▸; Blach *et al.*, 2020 ▸), we have developed the program package *MATSAS*. It allows the analysis of data obtained from small-angle and very small angle scattering of neutrons and X-rays [very small angle neutron scattering (VSANS), small-angle neutron scattering (SANS), wide-angle X-ray scattering (WAXS), ultra-small-angle X-ray scattering (USAXS) and small-angle X-ray scattering (SAXS)].

*MATSAS* analyses data from pinhole-geometry, time-of-flight (TOF) and Bonse–Hart machines and was tested using data acquired at FRM-II (Research Reactor Munich II, Garching, Germany) and ORNL (Oak Ridge National Laboratory, Tennessee, USA) (Rezaeyan, Pipich *et al.*, 2019*a* ▸,*b* ▸; Rezaeyan, Seemann *et al.*, 2019 ▸; Seemann *et al.*, 2019 ▸). *MATSAS* does post-processing of data obtained from research facilities. It is assumed that initial corrections for sample thickness, transmission, detector sensitivity, instrument background, multiple scattering and noise have been made using the instrument-specific settings at the facility itself, providing data in absolute units (Hinde, 2004 ▸; Melnichenko, 2015 ▸). *MATSAS* is primarily oriented towards the structural analysis of sedimentary rocks using a polydisperse spherical (PDSP) model. The *MATSAS* software is constantly refined to broaden its functionality, making it applicable to isotropic and partially ordered objects such as biological nanoparticle systems, colloidal solutions, and polymers in solution and bulk. It is an open-source computer tool for academic users and is freely available on GitHub (https://github.com/matsas-software/MATSAS). Open-source access reflects transparency in the fundamental assumptions and solving approaches employed in the program and allows third parties to interface their in-house programs with the data analysis framework of the program (Liu *et al.*, 2012 ▸) and to help in accelerating its development. In this paper, we summarize the main components of *MATSAS* and its development framework.

## 2. Program overview

*MATSAS* features a script-based package in MATLAB (The MathWorks Inc., Natick, MA, USA), which integrates computation and visualization in an easy-to-use environment. The *MATSAS* program is a versatile computer tool allowing both users and developers to add additional tools and develop specific novel applications. The flexible user-friendly framework of *MATSAS* for basic routines, such as intensity calculation or model alignment, allows anyone with basic programming skills to improve or adapt *MATSAS* to better reflect user-specific needs. Furthermore, the current version of the package includes the PDSP model to analyse SAS data in terms of theoretical intensity computation, the *f*(*r*) probability function of pore size distribution and model refinement. The PDSP model is the method commonly used for SAS analysis of a polydisperse system of randomly oriented independently scattering particles and is ubiquitous for fractal microstructures (*e.g.* sedimentary rocks) as well as other porous systems (Radlinski, Ioannidis *et al.*, 2004 ▸), provided that the particle-shape distribution is independent of the distribution of particle dimensions in the polydisperse system (Schmidt, 1982 ▸). The script-based *MATSAS* code allows parameters to be tuned for more features of each routine.

**Figure 2:**
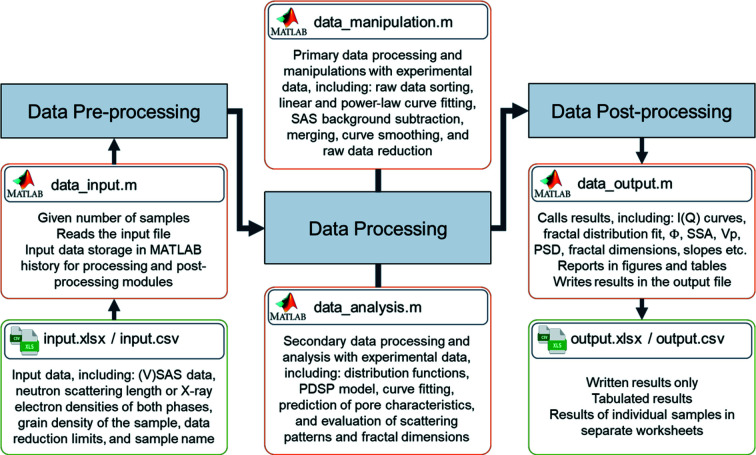
A schematic flow chart of *MATSAS* programs and their functionalities.

Use of the *MATSAS* program is divided into three steps: (i) pre-processing of raw or facility post-corrected SAS and very small angle scattering (VSAS) data as well as physical information, (ii) processing of the imported information to produce *I*(*Q*) versus *Q* curves, combine the SAS and VSAS curves, and fit the PDSP model, and (iii) post-processing to display and export structural information obtained from the samples being analysed. Fig. 2 ▸ illustrates the main components of the present version of *MATSAS*.

Detailed instructions on how to use the package are available on GitHub. Supporting information and command descriptions are embedded in each module. Errors and bugs can be invoked when no parameters or incorrect data are given to the command. We developed the package in Windows and recommend running it in Windows, Mac or Linux, with any Intel or AMD x86-64 processor with four logical cores and AVX2 instruction set support, as a minimum. Although the program runs satisfactorily without a specific graphics card, a hardware-accelerated graphics card supporting OpenGL 3.3 with 1 GB graphical processing unit (GPU) memory is recommended, as displaying figures and generating Microsoft *Excel* worksheets require more background processing.

## 3. Data pre-processing

The data pre-processing module is composed of two components: (i) data are prepared in a Microsoft *Excel* spreadsheet [*.xls(x)] or *.csv file, including (V)SAS data, neutron scattering length densities or X-ray electron densities of phases 1 and 2 (*e.g.* rock matrix and pore), grain density of the sample, data reduction limits (optional), and sample name, and (ii) the MATLAB data_input.m file reads and stores the imported data for the next step. *MATSAS* allows users to run a batch of samples. The units in the input files can be converted between different unit systems (between nm^−1^ and Å^−1^ for *Q*, for instance) by changing appropriate codes. The range(s) of data points can be adjusted for each data set individually or simultaneously for selected groups of files.

## 4. Data processing

The data processing module is used to manipulate and analyse the information imported. The primary data processing script is developed to manipulate scattering curves. The *data_manipulation.m* program carries out multiple tasks, including *I*(*Q*) data sorting, curve fitting, background subtraction, curve merging, curve smoothing and raw data reduction. The secondary data processing script file in *data_analysis.m* is designed to analyse *I*(*Q*)–*Q* curves and produce structural information. An arbitrary size distribution is created first and the PDSP model is then fitted to the processed scattering curve. Pore characteristics are predicted and fractal dimensions (including the pore fractal dimension, *D*_p_, surface fractal dimension, *D*_s_, and general fractal dimension, *D*_f_) are evaluated from the fit in this module.

### 4.1. Data manipulation

**Figure 3:**
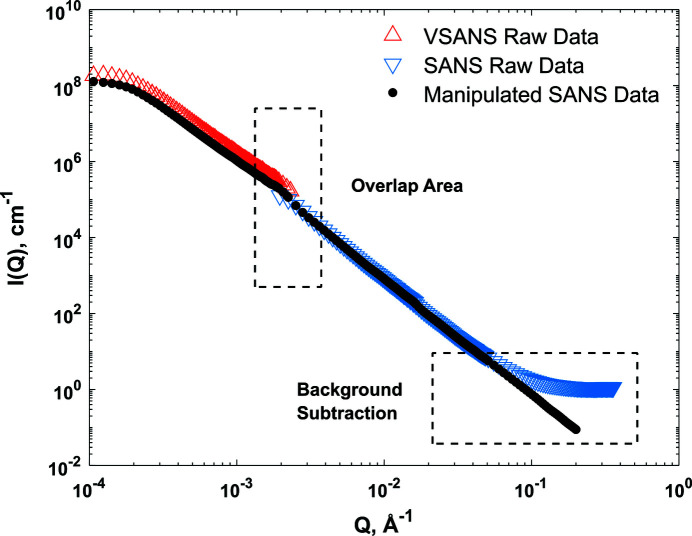
SANS data manipulated and processed on an arbitrary mudrock sample. Red, blue and black curves are the scattering profiles from the VSANS and SANS instruments and the net scattering after manipulation (merging, background subtraction and smoothing), respectively.

The program *data_manipulation.m* is a data processing module encompassing major SAS data processing steps for isotropic systems, from merging of scattering curves to background reduction. This program performs manipulations with one-dimensional data sets and calls other analysis and fitting programs via user-defined or built-in function files. The SAS data might have been collected at different sample-to-detector distances. Once data from several experimental curves have been combined for one specific instrument (*e.g.* SANS), they may not be sorted, which leads to numerical problems in further analysis. Data sorting is therefore carried out in the data manipulation package using a built-in function. If the SAS data consist of two scattering profiles obtained from two different instruments (*e.g.* VSANS and SANS), *MATSAS* allows users to merge the two curves using a least-squares fit in the overlapping range, as illustrated for example in Fig. 3 ▸. The SAS curve is the basis onto which the VSAS curve is rebinned. The high-*Q* background is subtracted using equation (3), 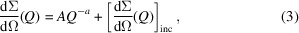
where the scattering varies with *Q*^−*a*^ in the high-*Q* limit before plateauing (Melnichenko, 2015 ▸). The value of the background 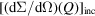 is determined from a linear plot of equation (4),
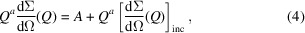
where 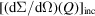 is the slope and *A* is the intercept (Melnichenko, 2015 ▸). Fig. 3 ▸ shows the background subtraction in the high-*Q* limit for a range that users can change manually in the program.

A noise-removal operation is embedded to remove the sparse data around the beam stop or detector edge. Raw data reduction, whose cut-off limits are determined in the data input files, is carried out as well. Two data smoothing operations are included in the package, which can be employed to obtain a smooth scattering profile for further structural analysis. Fractal dimensions and slope are determined here. For all operations, the propagation of uncertainty is performed using standard equations (Bevington & Robinson, 2003 ▸). A MATLAB plotting operation displays the currently active scattering profiles on a log–log scale. An advanced plotting option included in the plot permits users to change the plotting range, enlargement factor *etc.* The data manipulation file contains an output section, where the result of each operation can be used in subsequent data analysis. Information about the operation (type of operation, section names, functions, weights, ranges of points used *etc.*) is written in the package in green and allows modification or change of lines if needed.

### 4.2. Data analysis

The data analysis program calculates the intensity of SAS from a polydisperse system of scatterers (Porod, 1951 ▸, 1952 ▸; Guinier & Fournet, 1955 ▸). The intensity is expressed in terms of a fractal distribution of scatterers, also called the probability density of the pore size distribution *f*(*r*), for greater numerical stability (Ilavsky & Jemian, 2009 ▸). SAS curves from sedimentary rocks are usually linear on a log–log scale, particularly in the high-*Q* region, which reflects fractal behaviour (Melnichenko, 2015 ▸). Scattering from a fractal surface is equivalent to scattering from a system of polydisperse spherical scatterers (Schmidt, 1982 ▸), with a number–size distribution (the number of spheres with radii between *R* and *R* + d*R*) given by 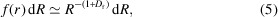
where *D*_f_ is the fractal dimension determined from the slope of the power-law scattering (Melnichenko, 2015 ▸). In practice, the distribution described in equation (5) and in the range *R*_min_ ≤ *R* ≤ *R*_max_ shows fractal behaviour between the upper and lower cut-off parameters. *f*(*r*) is expressed as 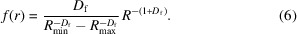
This is valid for *R*_max_ > *R*_min_ > 0 and *D*_f_ ∈ (−1, ∞), where *D*_f_ = 6 + slope. Scattering from a PDSP featured sample has a linear region with a similar slope −(1 + *D*_f_) and is described by (Radlinski, Ioannidis *et al.*, 2004 ▸)
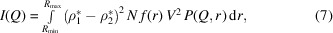
where 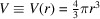 is the volume of a sphere of radius *r* (volume of a scatterer). In addition, *P*(*Q*, *r*) is the form factor of a sphere of radius *r* (Guinier & Fournet, 1955 ▸):
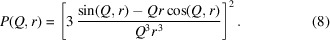
*N* is the total number of scatterers, which is related to the number size distribution as *N*(*r*) = *Nf*(*r*). *N*(*r*) is expressed as
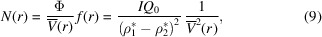
where 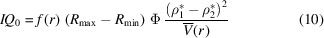
is the scattering intensity at *Q* = 0 and 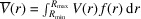 is the average volume of the scatterers (Radlinski *et al.*, 2002 ▸). Similarly to the approach of Ilavsky & Jemian (2009 ▸), *MATSAS* calculates equation (7) throughout the integration over a continuous size distribution with a summation over a discrete size histogram: 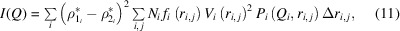
where the subscript *i* represents different scattering sizes and the subscript *j* describes bins in the size distribution. Δ*r*_*i*, *j*_ is the width of bin *j* and each scattering size has its own binning index *i*, *j*. *r* is the dimension of the scatterer (radius for spheres) and has limits 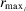 and 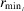. The radius *r* is calculated using *R* = 2π/*Q*, which is *R* = 2.5/*Q* in the fractal distribution (Radliński *et al.*, 2000 ▸).

*MATSAS* uses an arbitrary size distribution to model the scattering volume distribution *V*^2^(*r*)*P*(*Q*, *r*) and determine *f*(*r*). The user can change the theoretical ranges of the various size distributions in the data analysis program. Numerical calculations call limits on the range of dimensions (*r*_min_ and *r*_max_), the cut-off limits (*R*_min_ and *R*_max_) and the number of bins (*N*_bin_). This method results in a natural logarithmic step in dimension and uses three parameters, *R*_min_, *R*_max_ and *N*_bin_. The centres of the first (*r*_*i*, 1_) and last (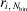) bins are *R*_max_ and *R*_min_, respectively, and extra fractional volumes are discarded for both bins: the volumes associated with 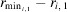 and 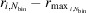 for the first and last bins, respectively. The widths of the bins are equal by selecting associated dimensions at regular increments of the cumulative distribution (Ilavsky & Jemian, 2009 ▸), leading to 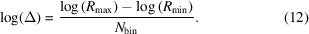
However, the numerical operation of the *data_analysis.m *file requires 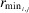, 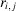, 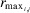, *f*_*i*_(*r*_*i*, *j*_) and 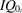 to fit the PDSP model in equation (7) to the measured *I*(*Q*) curve. The fitting procedure employs *f*(*r*) and *IQ*_0_ as fitting parameters for each iteration to attain a match where the summation of square errors (SSQ) tends to a minimum (Hinde, 2004 ▸).

To reduce the computation time taken by numerical integration, we found an analytical solution for the scattering volume distribution 
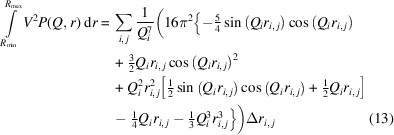
that transforms equation (11) into
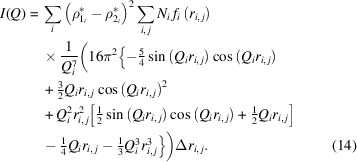


*MATSAS* simplifies the intensity calculation by substituting equation (9) into equation (14), leading to 
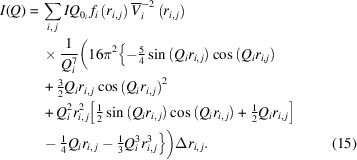


Once the match is reached, the data analysis program yields the structural characteristics of the scatterers using the fitted *f*(*r*) and *IQ*_0_ values. The specific surface area (SSA) of the scatterers is obtained following Hinde (2004 ▸): 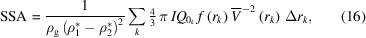
where the subscript *k* represents bins in the size distribution. The volume fraction of scatterers per unit volume (Φ) is calculated from equation (9), which results in 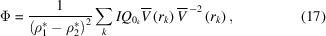
and the total volume of scatterers (*V*_p_) is obtained by
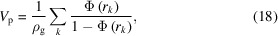
where the subscript *k* represents bins in the size distribution. Differential (d*V*/d*r* or d*A*/d**r**) and logarithmic differential (d*V*/dlog*r* or d*A*/dlog*r*) scatterer size distributions are calculated cumulatively (Meyer & Klobes, 1999 ▸).

The scattering intensity decays as *Q*^−*m*^ with different power-law exponents *m*; this indicates that *m* is related to the dimensionality of the pore as understood in terms of the concept of fractality (Mandelbrot, 1983 ▸). For a fractal pore scatterer, therefore, *D*_p_ = *m* with values 1 < *D*_p_ < 3, and for a surface fractal *D*_s_ = 6 − *m* with values 2 ≤ *D*_s_ ≤ 3 (Bale & Schmidt, 1984 ▸).

The scattering at different length scales indicates the Guinier, mass/pore fractal, surface fractal and Porod regions, suggesting that each fractal region is limited to a specific range of scattering vectors (Fritzsche *et al.*, 2016 ▸). Therefore, for sedimentary rocks *D*_p_ and *D*_s_ are geared to the ranges of 0.0003–0.003 cm^−1^ and 0.003–0.03 cm^−1^, respectively. In addition, *D*_f_ is included to reflect the fractality of the full pore system over the entire scattering vector range, *e.g.* 0.0003–0.03 cm^−1^ in sedimentary rocks (Rezaeyan, Pipich *et al.*, 2019*a* ▸,*b* ▸; Rezaeyan, Seemann *et al.*, 2019 ▸). These ranges can be changed by the user.

**Figure 4:**
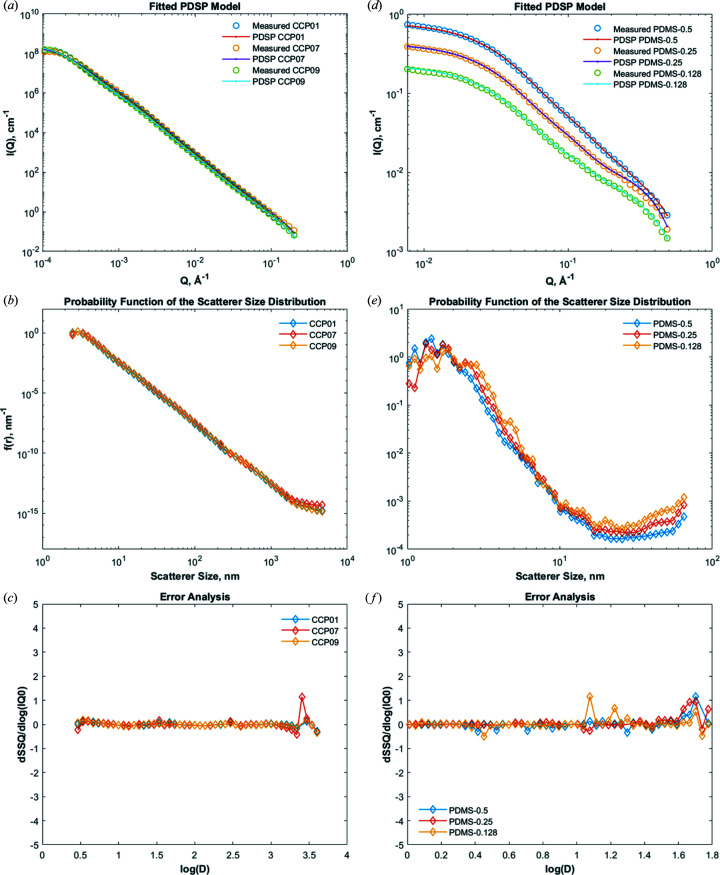
The PDSP model applied to SANS data obtained from three rock samples (Opalinus Clay) and three poly­di­methyl­siloxane (PDMS) polymers of volume fractions 0.128, 0.25 and 0.5 in toluene. Rock samples: (*a*) measured *I*(*Q*) curves after manipulation and *I*(*Q*) curves obtained from the PDSP model, (*b*) probability functions of the pore size distribution *f*(*r*), and (*c*) the error sensitivity dSSQ/dlog(*IQ*_0_) obtained after two iterations. PDMS samples: (*d*) the fitted PDSP model, (*e*) probability functions of the scatterer size distribution *f*(*r*) and (*f*) the error sensitivity obtained after 20 iterations.

For demonstration purposes, we tested the analysis operations on SANS and VSANS data obtained from three rock samples (Opalinus Clay) using batch mode. Opalinus Clay is a Jurassic mudrock that was obtained from the Mont Terri Underground Laboratory in Switzerland and has been described in detail previously (Busch *et al.*, 2017 ▸). Fig. 4 ▸(*a*) shows the PDSP modelled *I*(*Q*) curves and the measured *I*(*Q*) curves after two iterations of the fitting operation. The first iteration starts with initial guesses for *f*(*r*) and *IQ*_0_, which are obtained from the slope of the scattering curves and the Guinier & Fournet (1955 ▸) approximation, respectively. SSQ tends to a minimum after the second iteration; two iterations are recommended for most rock samples (Hinde, 2004 ▸). Fig. 4 ▸(*b*) shows *f*(*r*) after two iterations on a log–log scale. *f*(*r*) levels off at scatterer sizes ≳2 µm because the scattering intensities of large scatterers are smeared, possibly due to instrument artefacts at the edge of the detector. The error sensitivity, expressed as dSSQ/dlog(*IQ*_0_), relates SSQ to the number of iterations [Fig. 4 ▸(*c*)]. The value of dSSQ/dlog(*IQ*_0_) varies around zero for all scatterer sizes. However, as illustrated in Fig. 4 ▸(*c*), this can deviate where the fit is rather poor for large scatterer sizes (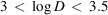) due to different instrument resolutions or noise within overlap areas. SSQ is magnified when the number of iterations exceeds two, resulting in an attenuation of *f*(*r*). Nevertheless, we recommend attaining a smooth *f*(*r*) if the optimum fit requires a larger number of iterations for a specific sample. χ^2^ tolerance can be used for the fit when the user has no preference for the number of iterations.

We also tested the PDSP model on three poly­di­methyl­siloxane (PDMS) polymers with volume fractions of 0.128, 0.25 and 0.5 in toluene to demonstrate the applicability of the fitting operation for a non-power-law nanostructure in solution [Figs. 4 ▸(*d*)–4 ▸(*f*)]. Fig. 4 ▸(*f*) displays the numerical flexibility of the fitting procedure after 20 iterations.

## 5. Data post-processing

**Figure 5:**
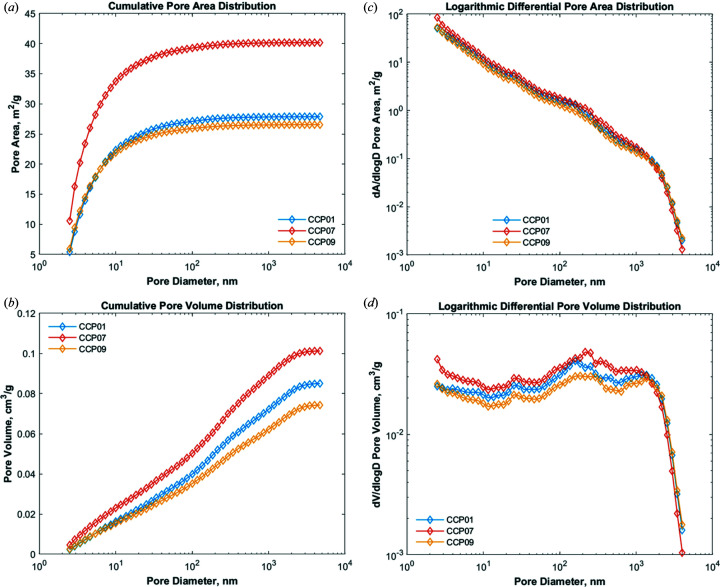
The PDSP model applied to SANS data obtained from three rock samples (Opalinus Clay). (*a*) Cumulative pore area distribution, (*b*) logarithmic differential pore area distribution, (*c*) cumulative pore volume distribution and (*d*) logarithmic differential pore volume distribution.

The data post-processing module is made of two components, including *data_output.m* in MATLAB and the results reported in figures and tabulated files. The *data_output.m* file calls the results of individual samples, reports the results in figures and tables in the MATLAB command window, and writes the results in output.xlsx. The results include measured, processed and predicted scattering curves, fractal distribution fit (*f_r_*), specific surface area (SSA), porosity (Φ), pore volume (*V*_p_), pore size distribution (PSD) by pore volume or pore area, fractal dimensions, slopes of scattering curves, pore characteristics divided into macro-, meso- and micropores, and background subtraction values. Some results for the Opalinus Clay samples are shown in Fig. 5 ▸ and Table 2 ▸. The results for individual samples are produced and saved in figure formats (*.tif and *.emf), *Excel* spreadsheets and .csv files for use in further specific analyses. The results are usable if the raw SAS data are provided in absolute units; otherwise users must report pore characteristics in arbitrary units.

## 6. Conclusions

*MATSAS* encompasses a set of modules allowing for a full analysis of (V)SANS and (V)SAXS data from porous systems, *e.g.* sedimentary rocks. *MATSAS* is written in MATLAB and combines a desktop environment tuned for data processing and structural analyses with pre- and post-processing modules. The pre-processing module is used to import data from Microsoft *Excel* spreadsheets or .csv files into MATLAB. The main module performs data manipulation and analysis in which *I*(*Q*)–*Q* curves are processed and the PDSP model is fitted to produce structural information for porous systems. The post-processing module displays results in the form of tables and figures and exports them in Microsoft *Excel* spreadsheets or .csv files. *MATSAS* is the first SAS program that provides a full suite of pore characterizations. The programs included in *MATSAS* are publicly available on GitHub (https://github.com/matsas-software/MATSAS) for academic users.

## Figures and Tables

**Table 1:** Common SAS programs and their capabilities and applicabilities

SAS program	Capabilities	Applicability	Reference
*FIT2D*	2D image data reduction/manipulation and peak fitting		Hammersley (1995 ▸, 2016 ▸)
*BerSANS*	Data acquisition/reduction		Keiderling (1997 ▸)
*DALAI_GA*	*Ab initio* shape determination	Biological systems	Chacón *et al.* (1998 ▸)
*FISH*	Peak analysis and parametric fitting using various form and structure factors		Heenan (1999 ▸)
*SAX3D*	*Ab initio* shape determination	Biological systems	Walther *et al.* (2000 ▸)
*SAXS/WAXS* software system	Data acquisition/reduction		Homan *et al.* (2001 ▸)
*GRASP*	Data acquisition/reduction		Dewhurst (2002 ▸)
*SAXSANA*	Data reduction, *Q* determination, data conversion, data correction, analysis of time-resolved data and data extrapolation	Biological systems	Hiragi *et al.* (2003 ▸)
*PRINSAS* †	Fitting of 1D curves using a spherical form factor for a polydisperse scattering system	Porous systems	Hinde (2004 ▸)
*ATSAS*	Data reduction, data processing and 3D modelling	Biological systems	Konarev *et al.* (2006 ▸), Petoukhov *et al.* (2012 ▸), Franke *et al.* (2017 ▸), Manalastas-Cantos *et al.* (2021 ▸)
*DAMMIF*	*Ab initio* shape determination for disordered systems and solutions	Nanostructures	Franke & Svergun (2009 ▸)
*IRENA* †	Plotting SAS data, merging of two overlapping data sets, and fitting form and structural models to data from contrast variation experiments	A wide range of systems	Ilavsky & Jemian (2009 ▸)
*BioXTAS RAW*	Isotropic SAXS data reduction, primary data analysis and calculations of the pair-distance distribution functions, averaging, subtraction and analysis of radius of gyration and molecular weight, calculation of inverse Fourier transforms and envelopes, processing of inline size-exclusion chromatography coupled SAXS data, and data deconvolution	Biological systems	Nielsen *et al.* (2009 ▸), Hopkins *et al.* (2017 ▸)
*SCATTER*	2D data analysis	Nano- and mesoscale oriented structures	Förster *et al.* (2010 ▸)
*SAAF*	SANS data analysis using a set of standard models	Polymers	Zhao (2011 ▸)
*SASTBX*	Data reduction, model reconstruction, model refinement and shape retrieval	Biological systems	Liu *et al.* (2012 ▸)
*SASET*	1D and 2D data analysis and fitting of data using scattering models and anisotropy methods	Anisotropic structures	Muthig *et al.* (2013 ▸)
*MolScat* and *SAFIR*	Modelling of 3D macromolecular structures	Biological systems	Hofmann & Whitten (2014 ▸)
*SASfit*	Reduction of oversampled data sets, confidence assessment of optimized model parameters and availability of custom user-provided models	Polymers	Breßler *et al.* (2015 ▸)
*QtiSAS*/*QtiKWS*†	Graphical visualization, reduction, analysis and fitting of data using various scattering models	A wide range of systems	https://www.qtisas.com/
*SASview* †	Data reduction, manipulation and analysis using several form and structure factors with polydispersity and orientational distributions	A wide range of systems	http://www.sasview.org/

† *PRINSAS*, *IRENA*, *QtiSAS* and *SASview* are commonly used for analysis of SAS data obtained from porous systems featuring a wide range of pore sizes.

**Table:** The subscripts meso and macro represent properties in meso- and macropore sizes, respectively.

Sample ID	*m*	*D*_f_	*D*_s_	*D*_p_	*I*_BG VSAS_ (cm^−1^)	*I*_BG SAS_ (cm^−1^)	SSA (m^2^ g^−1^)	SSA_macro_ (m^2^ g^−1^)	SSA_meso_ (m^2^ g^−1^)
CCP01	−3.06	2.94	2.88	2.84	15317	1.15	31.6	1.4	30.2
CCP07	−3.05	2.95	2.88	2.76	7032	1.23	44.8	1.6	43.1
CCP09	−3.07	2.93	2.88	2.86	14818	0.92	29.6	1.2	28.5

**Table:** 

Sample ID	*V*_p_ (cm^3^ g^−1^)	*V*_macro_ (cm^3^ g^−1^)	*V*_meso_ (cm^3^ g^−1^)	Φ (%)	Φ_macro_ (%)	Φ_meso_ (%)	SSQ	χ^2^
CCP01	0.0880	0.0541	0.0339	23.7	14.6	9.1	0.01	0.003
CCP07	0.1036	0.0601	0.0435	28.0	16.3	11.8	0.09	0.006
CCP09	0.0773	0.0472	0.0301	21.1	12.9	8.2	0.01	0.005
